# Behavioral and social pathways of adolescent drug use in Indonesia: implications for community-based prevention

**DOI:** 10.1186/s12889-026-26667-z

**Published:** 2026-02-21

**Authors:** Muhammad Azwar, Dian Furqani Hamdan, Ratnasari Iskandar, Lilis Widiastuty, Yudi Adnan

**Affiliations:** 1 Department of Public Health, Universitas Mega Buana Palopo, Palopo, South Sulawesi Indonesia; 2 Nursing Profession Program, Universitas Mega Buana Palopo, Palopo, South Sulawesi Indonesia; 3 Department of Public Health, Universitas Alauddin, Makassar, South Sulawesi Indonesia

**Keywords:** Adolescent drug use, Behavioural pathways, Self-regulation, Behavioural intention, Community-based prevention

## Abstract

**Background:**

Adolescent drug use remains a major public health concern, particularly in low- and middle-income countries where rapid social change affects family and community structures. Most existing studies examine behavioural and social determinants as independent predictors, offering limited understanding of the pathways through which social environments influence adolescent substance-use behaviour.

**Methods:**

A cross-sectional analytical study was conducted among 350 adolescents aged 15–19 years recruited from secondary schools and community youth settings in Indonesia. Behavioural and social constructs including family support, parental monitoring, community engagement, risk perception, self-regulation, behavioural intention, and adolescent drug use were measured using validated multi-item scales. Data were analysed using Partial Least Squares Structural Equation Modelling (PLS-SEM) to estimate direct and indirect relationships among constructs.

**Results:**

Family support (β = 0.29, *p* < 0.001) and parental monitoring (β = 0.34, *p* < 0.001) were positively associated with adolescent self-regulation. Community engagement showed a positive association with risk perception (β = 0.27, *p* < 0.001). Risk perception (β = −0.24, *p* = 0.001) and self-regulation (β = −0.28, *p* < 0.001) were inversely associated with adolescent drug use, indicating protective effects. Behavioural intention emerged as the strongest and most proximal predictor of drug use (β = 0.45, *p* < 0.001). Overall, the findings support a pathway-based model in which family and community contexts influence adolescent drug use indirectly through cognitive and self-regulatory mechanisms.

**Conclusions:**

Adolescent drug use in Indonesia is shaped by interconnected behavioural and social pathways rather than isolated risk factors. Prevention strategies should therefore prioritise strengthening self-regulation and addressing behavioural intentions through family- and community-based interventions. These findings provide empirical support for behaviourally grounded and contextually relevant community-based prevention programmes.

**Graphical Abstract:**

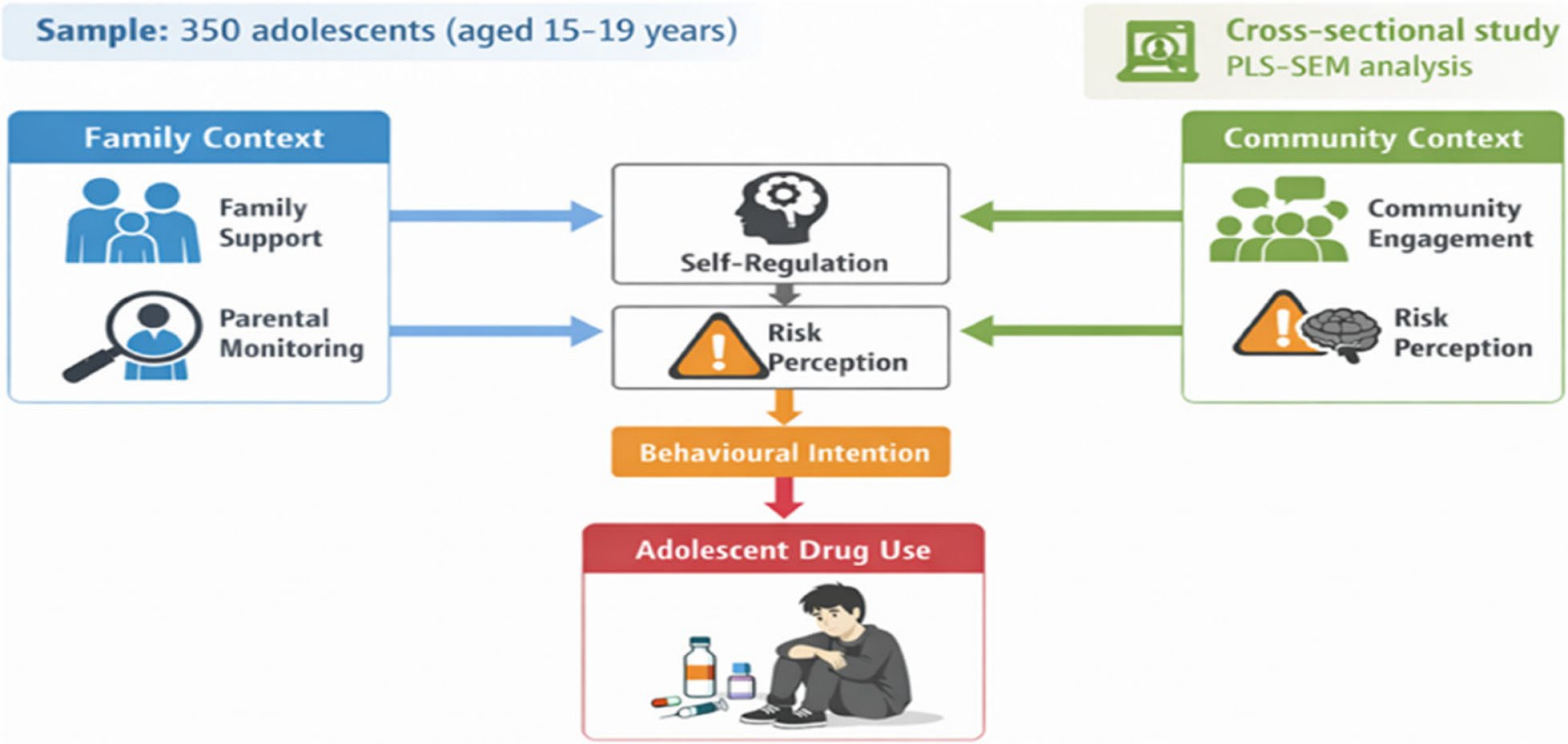

**Supplementary Information:**

The online version contains supplementary material available at 10.1186/s12889-026-26667-z.

## Introduction

Adolescent drug use remains a major public health concern globally, contributing to a wide range of adverse health, social, and developmental outcomes [1, 2]. Early initiation of substance use is associated with increased risks of mental health disorders, educational disengagement, and sustained substance dependence into adulthood [3, 4]. In many low- and middle-income countries, these risks are amplified by rapid social change, shifting norms, and unequal access to effective prevention resources, underscoring the need for evidence-based and context-sensitive prevention strategies [5].

Existing literature has consistently shown that adolescent drug use is shaped by multiple behavioural and social determinants, including family dynamics, peer environments, risk perception, and broader community contexts [6, 7]. Behavioural and social learning theories further suggest that adolescents’ substance-related behaviours do not develop in isolation, but are formed through continuous interaction with their social environments, observation of role models, and reinforcement of perceived norms [8, 9]. Empirical studies across diverse cultural and socioeconomic settings have identified peer substance use, weak parental monitoring, and limited social support as key correlates of adolescent drug use, underscoring the interconnected influence of individual cognition and social context on risk behaviour [10, 11].

In Indonesia, drug use among adolescents and young people remains a public health concern. The National Narcotics Board (BNN) reported that the prevalence of drug use in the student and university sector was 3.2%, equivalent to approximately 2.3 million individuals, based on national survey findings [12]. Beyond these numbers, community settings such as neighbourhood circles, youth groups, and peer social spaces can influence how young people spend their time, who they interact with, and what behaviours are considered acceptable. These social influences do not operate solely through direct supervision or control, but also through adolescents’ internal views about what is risky, what is normal, and how they manage impulses and decision-making.

These processes occur within Indonesia’s sociocultural context, where strong family cohesion, religious and cultural norms, and informal community social control may shape adolescents’ opportunities for supervision and social learning. At the same time, rapid urbanisation and evolving youth practices, including peer-driven social activities and digital media exposure, may increase adolescents’ access to risk-promoting influences and weaken some traditional protective boundaries. For this reason, understanding adolescent drug use in Indonesia requires a pathway-based approach that connects family and community environments with adolescents’ cognitive processes and self-regulation before substance use occurs.

However, much of the existing evidence conceptualises behavioural and social determinants of adolescent drug use as independent or linear predictors, providing limited insight into the mechanisms through which social environments are translated into individual substance-use behaviours [13, 14]. This limitation is particularly evident in low- and middle-income country settings, where social structures, family dynamics, and community influences differ substantially from those typically examined in high-income contexts. In Indonesia, adolescent drug use has emerged as an increasingly important public health issue amid rapid demographic transition, urbanisation, and changing patterns of youth social interaction. National reports and local studies have documented prevalence trends and selected risk factors; however, this evidence remains largely descriptive and fragmented, with few studies examining how behavioural and social determinants interact as part of an integrated process shaping adolescent drug use [15–17]. As a result, there is a critical lack of pathway-based evidence capable of informing community-based prevention strategies that move beyond isolated risk factors toward interventions grounded in behavioural mechanisms.

Conceptual framework. This study is guided by an integrative pathway perspective in which social contexts influence adolescent drug use through cognitive and behavioural mechanisms. Specifically, we hypothesised that family-related factors (family support and parental monitoring) strengthen adolescents’ self-regulation by providing emotional security, behavioural guidance, and consistent supervision. We further hypothesised that community engagement enhances adolescents’ risk perception by increasing exposure to prosocial norms, prevention-related information, and structured social participation. Finally, cognitive processes reflected in risk perception and self-regulation were expected to shape behavioural intention, which functions as the most proximal determinant of adolescent drug use behaviour.

Therefore, this study aimed to examine and model behavioural and social pathways of adolescent drug use in Indonesia using Partial Least Squares Structural Equation Modelling (PLS-SEM) based on data from 350 adolescents aged 15–19 years. The study tested the following hypotheses: (H1) family support is positively associated with adolescent self-regulation; (H2) parental monitoring is positively associated with adolescent self-regulation; (H3) community engagement is positively associated with adolescent risk perception; (H4) self-regulation is inversely associated with adolescent drug use; (H5) risk perception is inversely associated with adolescent drug use; and (H6) behavioural intention is positively associated with adolescent drug use.

This study makes three main contributions to the literature. First, it advances theoretical understanding by framing adolescent drug use as a pathway-driven phenomenon shaped by interacting behavioural and social mechanisms. Second, it contributes methodologically by applying PLS-SEM to capture complex, multidirectional relationships among determinants of adolescent drug use in a low- and middle-income country context [18, 19]. Third, it provides empirical evidence to inform the design of community-based prevention strategies that are behaviourally grounded and contextually relevant, thereby strengthening the scientific basis for preventive public health interventions targeting adolescents.

## Materials and methods

### Study design and setting

This study employed a cross-sectional analytical design [20] to examine behavioural and social pathways of adolescent drug use in Indonesia. A cross-sectional approach was considered appropriate for modelling complex interrelationships among multiple behavioural and social constructs at a single point in time, particularly for pathway-based exploratory analysis. The study was conducted in selected secondary schools and community youth settings located in urban and peri-urban areas. Data were collected between January and March 2025. These settings were chosen to capture variation in social environments and community contexts relevant to adolescent development and substance-use behaviour. The analytical framework was explicitly designed to move beyond the identification of isolated risk factors and instead to examine indirect and mediated relationships among family, community, cognitive, and behavioural variables using Structural Equation Modelling with a Partial Least Squares approach (PLS-SEM) [21].

### Study population and sample

The study population consisted of adolescents aged 15–19 years, corresponding to mid-to-late adolescence, a developmental period characterised by increased autonomy, heightened peer influence, and vulnerability to risk-taking behaviours. A total of 350 adolescents were included in the final analysis. The sample size exceeded the minimum requirements for Partial Least Squares Structural Equation Modelling (PLS-SEM). Sample adequacy was assessed using the 10-times rule, based on the maximum number of structural paths directed at any latent construct in the model, as well as statistical power considerations for detecting medium effect sizes. This sample size was therefore sufficient to ensure stable parameter estimation and adequate statistical power. Participants were recruited using a multistage sampling approach. In the first stage, secondary schools [22] and community youth centres were purposively selected to reflect diverse socio-demographic characteristics and residential contexts. In the second stage, eligible adolescents were selected using stratified random sampling based on age and sex to ensure balanced representation across key demographic groups.

### Eligibility criteria

Adolescents were eligible to participate if they: (1) were aged 15–19 years; (2) were recruited from the selected secondary school or community youth settings; (3) were able to understand and complete the questionnaire; and (4) provided written informed consent prior to participation. Adolescents were excluded if they: (1) declined to participate or withdrew at any stage; (2) submitted incomplete questionnaires with substantial missing responses; or (3) did not provide the required parental/legal guardian consent when applicable.

### Data collection procedures

Data were collected using a structured, self-administered questionnaire. The instrument was developed based on established behavioural and social theories and informed by prior empirical studies on adolescent substance use. Trained research assistants administered the survey following standardised procedures to minimise information bias and ensure consistency across study sites. To protect participants’ privacy, questionnaires were completed anonymously, and no personally identifiable information was collected. Written informed consent was obtained from all participants prior to data collection. For participants under 18 years of age, additional consent was obtained from parents or legal guardians. All participants were informed that their participation was voluntary and that they could withdraw at any time without any consequences.

### Ethical considerations

Ethical approval for this study was obtained from the Research Ethics Committee of the Mitra Husada Foundation, Indonesia. The study procedures were conducted in accordance with the Declaration of Helsinki and relevant national guidelines. Informed consent was obtained from all participants before data collection. For participants aged under 16 years, written informed consent was obtained from their parents or legal guardians, and assent was obtained from the adolescents. Participation was voluntary, and all responses were kept confidential and analysed anonymously.

### Measures

Adolescent drug use was specified as a latent construct measured using multiple indicators reflecting lifetime and recent use of illicit substances. Behavioural constructs included risk perception, self-regulation, and behavioural intention related to substance use. Social constructs comprised family support, parental monitoring, community engagement, and peer influence. All constructs were measured using multi-item scales adapted from previously validated instruments, with responses recorded on five-point Likert scales ranging from strongly disagree to strongly agree. Prior to the main survey, the questionnaire was pilot-tested among adolescents with similar characteristics to assess clarity, cultural appropriateness, and internal consistency. Minor revisions were made based on feedback from the pilot test. Peer influence was conceptualised as a contextual social construct, reflecting adolescents’ exposure to peer environments, rather than as a direct behavioural determinant. This decision was made to align with the pathway-based analytical perspective, which emphasises indirect social influences operating through cognitive and behavioural mechanisms. Each construct was measured using multiple items adapted from previously validated instruments and contextualised to the adolescent setting. Higher scores reflected greater levels of the underlying construct (e.g., stronger parental monitoring, higher risk perception, or stronger behavioural intention). Prior to data collection, the instrument was pilot-tested to ensure clarity and cultural appropriateness, and internal consistency reliability was assessed during measurement model evaluation.

### Partial least squares structural equation modelling

Data were analysed using Partial Least Squares Structural Equation Modelling (PLS-SEM) with SmartPLS version 4.0.8.2. PLS-SEM was selected due to its suitability for exploratory and pathway-based modelling, its emphasis on prediction-oriented objectives, and its robustness to non-normal data distributions and complex model structures. The assumed causal ordering followed a pathway logic in which upstream social contexts (family support, parental monitoring, and community engagement) influence intermediate cognitive and self-regulatory mechanisms (risk perception and self-regulation), which in turn shape proximal behavioural determinants (behavioural intention) and ultimately adolescent drug use. The analysis followed a two-stage procedure. First, the measurement model was evaluated to assess indicator reliability, internal consistency reliability, convergent validity, and discriminant validity. Indicator reliability was examined using outer loadings, with values of 0.70 or higher considered acceptable. Internal consistency reliability was assessed using composite reliability (CR), while convergent validity was evaluated using the average variance extracted (AVE), with values of 0.50 or higher indicating adequate convergence. Discriminant validity was examined using the heterotrait–monotrait (HTMT) ratio. Second, the structural model was assessed by examining collinearity among constructs using variance inflation factor (VIF) values, followed by estimation of structural path coefficients. Model performance was evaluated using coefficients of determination (R²), effect sizes (f²), and predictive relevance (Q²). The statistical significance of structural relationships was assessed using a bootstrapping procedure with 5,000 resamples, applying a two-tailed significance level of *p* < 0.05.

### Data analysis

Descriptive statistics were generated using SPSS to summarise socio-demographic characteristics of participants. All PLS-SEM analyses were conducted using SmartPLS. Missing data were minimal (< 5%) and were handled using mean replacement procedures, which are considered acceptable in PLS-SEM when the proportion of missing data is low and missingness is random.

## Results

Table 1 summarises the socio-demographic characteristics of the participants (*n* = 350), including age, sex, educational level, school attendance, place of residence, living arrangement, parental education and employment status, household income, and participation in community or youth activities.

**Table 1:** Socio-demographic characteristics of study participants (*n* = 350)

Characteristic	Category	*n*	%
Age (years)	15–16	118	33.7
	17–18	162	46.3
	19	70	20.0
Sex	Male	176	50.3
	Female	174	49.7
Educational level	Junior secondary school	132	37.7
	Senior secondary school	218	62.3
School attendance status	Currently enrolled	331	94.6
	Not currently enrolled	19	5.4
Place of residence	Urban	201	57.4
	Peri-urban	149	42.6
Living arrangement	Living with both parents	214	61.1
	Living with one parent	87	24.9
	Living with relatives/others	49	14.0
Parental education (highest)	Primary or less	96	27.4
	Secondary	172	49.1
	Tertiary	82	23.4
Parental employment status	At least one parent employed	297	84.9
	Both parents unemployed	53	15.1
Monthly household income	Below regional minimum wage	143	40.9
	At or above regional minimum wage	207	59.1
Participation in community or youth activities	Yes	219	62.6
	No	131	37.4

Table 2 presents the measurement model evaluation results, including outer loadings, composite reliability (CR), and average variance extracted (AVE) for each construct. Discriminant validity was assessed using the heterotrait–monotrait (HTMT) ratio.

**Table 2:** Measurement model evaluation: indicator reliability, internal consistency, and convergent validity

Construct	Indicator	Outer loading	Composite reliability (CR)	Average variance extracted (AVE)
Peer influence	PI1	0.81	0.86	0.60
	PI2	0.84		
	PI3	0.76		
	PI4	0.71		
Family support	FS1	0.88	0.88	0.66
	FS2	0.82		
	FS3	0.74		
Parental monitoring	PM1	0.89	0.90	0.69
	PM2	0.83		
	PM3	0.76		
Community engagement	CE1	0.85	0.87	0.64
	CE2	0.78		
	CE3	0.72		
Risk perception	RP1	0.86	0.89	0.68
	RP2	0.81		
	RP3	0.75		
Self-regulation	SR1	0.87	0.91	0.65
	SR2	0.83		
	SR3	0.78		
	SR4	0.73		
Behavioural intention	BI1	0.90	0.90	0.71
	BI2	0.84		
	BI3	0.77		
Adolescent drug use	DU1	0.92	0.92	0.75
	DU2	0.86		
	DU3	0.79		

Table 3 presents the structural model estimates across all study constructs. Family support and parental monitoring were linked to self-regulation, while community engagement was linked to risk perception. In addition, risk perception and self-regulation showed negative relationships with adolescent drug use, whereas behavioural intention showed a positive relationship. The corresponding path coefficients (β), t-values, p-values, and effect sizes are reported in Table 3.

**Table 3:** Structural model results for behavioural and social pathways of adolescent drug use

Structural relationship	Path coefficient (β)	t-value	*p*-value	Effect size (f²)
Family support and self-regulation	0.29	5.17	< 0.001	0.15
Parental monitoring and self-regulation	0.34	6.11	< 0.001	0.19
Community engagement and risk perception	0.27	3.95	< 0.001	0.11
Risk perception and adolescent drug use	−0.24	3.34	0.001	0.17
Self-regulation and adolescent drug use	−0.28	5.21	< 0.001	0.21
Behavioural intention and adolescent drug use	0.45	8.74	< 0.001	0.33

**Fig. 1:**
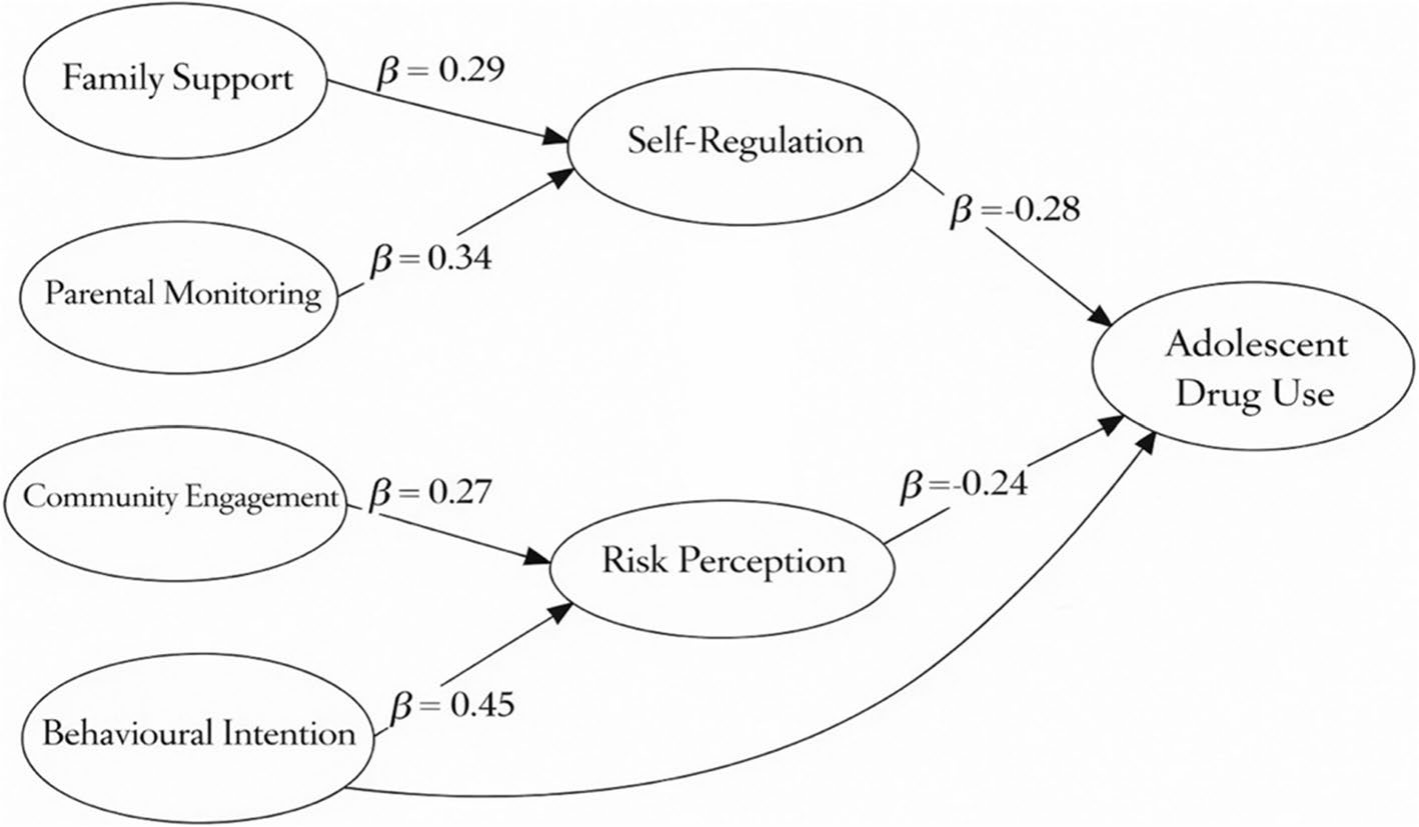
PLS-SEM Structural Model of Adolescent Drug Use

Figure 1. Structural model results based on PLS-SEM, showing the estimated path coefficients (β) among family support, parental monitoring, community engagement, self-regulation, risk perception, behavioural intention, and adolescent drug use.

## Discussion

This study provides three key contributions to the literature on adolescent substance use. First (theoretical contribution), it strengthens pathway-based understanding by demonstrating that family and community contexts shape adolescent drug use primarily through intermediate cognitive and self-regulatory mechanisms rather than isolated direct effects. Second (methodological contribution), it applies a PLS-SEM pathway framework to simultaneously estimate direct and indirect associations among multiple behavioural and social constructs within a low- and middle-income country context. Third (practical contribution), the findings inform community-based prevention by identifying behavioural intention, self-regulation, and risk perception as priority leverage points for intervention design, with families and communities serving as key upstream contexts for strengthening protective mechanisms.

The socio-demographic characteristics of the study population provide an important context for interpreting these findings. The predominance of mid-to-late adolescents reflects a developmental stage characterised by increasing autonomy, heightened responsiveness to peer norms, and ongoing maturation of behavioural control. The balanced sex distribution suggests that the identified pathways are not gender-specific but represent general behavioural processes during adolescence [23]. High levels of school enrolment and participation in community activities indicate that most adolescents were embedded within structured social environments, underscoring the relevance of family- and community-based mechanisms in shaping substance-use behaviour [24].

Family support and parental monitoring were both associated with stronger adolescent self-regulation, highlighting the family environment as a critical upstream determinant of behavioural control. Consistent with previous studies and meta-analytic evidence, stronger parental monitoring is linked to better impulse regulation and safer behavioural decision-making, which may in turn reduce adolescents’ vulnerability to substance use. Supportive family relationships may foster emotional security and adaptive coping, while consistent parental monitoring provides external structure that adolescents gradually internalise [25]. These findings are consistent with developmental and social learning perspectives, which emphasise that self-regulation emerges through sustained interaction between relational support and behavioural guidance.

Importantly, family-related factors did not exhibit direct associations with adolescent drug use in the present model. Unlike prior work that often conceptualises family variables as direct buffers against substance use [26, 27], the present pathway-based model highlights that family contexts may reduce drug use indirectly by strengthening adolescents’ self-regulatory capacity. Instead, their influence was mediated through self-regulation, indicating that family contexts shape adolescents’ behavioural capacity rather than directly constraining substance use [28]. This pathway-based interpretation refines prior research that often conceptualises family support and monitoring as direct protective factors, by demonstrating the mechanisms through which these influences operate. In settings where family cohesion remains culturally salient, such as Indonesia, strengthening self-regulatory capacity may therefore represent a particularly effective prevention leverage point [29, 30].

Community engagement was positively associated with adolescents’ risk perception, suggesting that involvement in community or youth activities enhances awareness of substance-related risks. This is consistent with evidence that community-based participation can reinforce protective norms and increase awareness of health risks among adolescents [31, 32]. Community contexts may function as informal learning environments in which norms, values, and health-related information are reinforced through social interaction. Participation in structured activities may also increase exposure to role models and peer groups that promote health-protective behaviours, thereby strengthening cognitive appraisal of risk [33].

However, community engagement did not show a direct association with drug use, underscoring the importance of intermediate cognitive mechanisms in the pathway model. In contrast to studies that interpret community involvement as directly protective [34], the present findings indicate that community engagement may influence substance use only when it translates into strengthened cognitive processes such as risk perception. This finding indicates that community-based prevention efforts are most effective when they explicitly enhance adolescents’ understanding of substance-related risks, rather than assuming that participation alone is sufficient to influence behaviour.

Risk perception and self-regulation were both inversely associated with adolescent drug use, reinforcing their importance as core protective mechanisms within the behavioural pathway. This pattern is consistent with behavioural and health decision-making perspectives in which higher perceived risk and stronger self-regulatory skills are associated with a lower likelihood of engaging in substance-use behaviour [35, 36]. Adolescents who perceived higher risks and who demonstrated stronger self-regulatory capacities were less likely to engage in substance use [37]. These findings align with health behaviour theories that emphasise the combined influence of cognitive evaluation and behavioural control [38]. Nevertheless, the moderate magnitude of these associations suggests that awareness and regulation alone may be insufficient in contexts where motivational drivers are strong [39, 40].

Behavioural intention emerged as the strongest predictor of adolescent drug use, underscoring its central role in translating psychosocial influences into behaviour. This finding is consistent with intention-based behavioural theories, which position intention as the most proximal antecedent of action [41]. This finding supports theoretical models that position intention as the immediate precursor to action, shaped by social norms, attitudes, and perceived acceptability of substance use. Even in the presence of protective factors such as high risk perception or strong self-regulation, favourable intentions toward drug use substantially increased the likelihood of engagement. This underscores the importance of prevention strategies that directly address adolescents’ motivational orientations and normative beliefs.

Overall, our findings align with prior research highlighting the importance of family and community contexts in adolescent substance use; however, they extend the literature by clarifying that these contextual factors may operate primarily through indirect behavioural pathways rather than direct associations. This pathway-based interpretation helps reconcile mixed findings across contexts and provides more actionable targets for prevention design.

Taken together, the findings support an integrated pathway model in which family and community contexts influence adolescent drug use indirectly through cognitive and behavioural mechanisms [42]. Family support and parental monitoring strengthen self-regulation, community engagement enhances risk perception, and these processes jointly reduce drug use, while behavioural intention operates as a critical proximal pathway that can either reinforce or undermine these protective influences [43].

From a practical prevention perspective, the findings suggest that community-based adolescent drug prevention programmes should move beyond general awareness campaigns by targeting behavioural intention and strengthening self-regulation skills through structured activities. Family-based components may prioritise improving parental monitoring practices and supportive communication that reinforces behavioural boundaries while fostering adolescents’ capacity for self-control. Community engagement strategies may integrate youth-led activities, peer mentoring, and safe extracurricular spaces that reinforce risk awareness and promote prosocial norms. Future studies should employ longitudinal designs to test temporal ordering of the pathways and intervention studies to evaluate whether strengthening these intermediate mechanisms leads to sustained reductions in adolescent drug use.

### Limitations

Several limitations of this study should be acknowledged. First, the cross-sectional design restricts the ability to establish temporal ordering and causal relationships among behavioural, cognitive, and social constructs. Although the proposed pathway-based model was grounded in established theory, the observed associations should be interpreted as correlational rather than causal.

Second, the study relied on self-reported survey measures, which may be subject to recall bias and social desirability bias, particularly for sensitive behaviours such as substance use. While anonymity and standardised data collection procedures were implemented to minimise reporting bias, the possibility of misclassification cannot be entirely excluded.

Third, the study was conducted within a specific Indonesian sociocultural context characterised by strong family cohesion, community norms, and evolving youth practices. Consequently, the generalisability of the findings to other cultural or national settings may be limited, and caution is warranted when extrapolating the results beyond similar contexts.

Finally, although the sample size was sufficient for PLS-SEM analysis, the study was not designed to examine subgroup differences across regions or socioeconomic strata. Future research should employ longitudinal designs to assess temporal pathways, integrate multi-method measurement approaches, and replicate the proposed model in different countries or populations to strengthen causal inference and external validity.

## Conclusion

This study shows that adolescent drug use in Indonesia cannot be adequately explained by single or isolated risk factors. Instead, substance-use behaviour emerges through interconnected behavioural and social pathways. By applying a pathway-based Structural Equation Modelling approach, this research demonstrates that family and community contexts influence adolescent drug use primarily through cognitive and self-regulatory processes. Supportive family environments and effective parental monitoring contribute to stronger self-regulation, while engagement in community activities is associated with higher risk perception. Among all constructs examined, behavioural intention represents the most immediate determinant of drug use, indicating its central role in converting social and cognitive influences into actual behaviour.

These findings contribute to the literature by moving beyond linear, factor-based explanations and providing empirical support for a mechanism-oriented understanding of adolescent drug use, particularly within a low- and middle-income country context. From a practical perspective, the results suggest that prevention efforts should not rely solely on information dissemination or normative appeals, but should focus on strengthening adolescents’ self-regulatory capacities and addressing behavioural intentions within family and community settings. Although the cross-sectional nature of the study limits causal interpretation, the findings offer a strong empirical basis for future longitudinal and intervention-based research aimed at developing and testing community-based prevention strategies grounded in behavioural mechanisms.

##  Supplementary Information

Supplementary Material 1. Table S1. Measurement constructs, indicator codes, and response scale.

## Data Availability

The datasets generated and/or analysed during the current study are not publicly available due to ethical and confidentiality considerations but are available from the corresponding author on reasonable request.

## Abbreviations

PLS-SEM: Partial Least Squares Structural Equation Modelling
CR: Composite Reliability
AVE: Average Variance Extracted
HTMT: Heterotrait-Monotrait Ratio
VIF: Variance Inflation Factor
