# Supplementary material for: Behavioral and social pathways of adolescent drug use in Indonesia: implications for community-based prevention
Source: BMC Public Health. 2026 Feb 21;26:1031. doi: 10.1186/s12889-026-26667-z (PMC13032467; doi:10.1186/s12889-026-26667-z)
Supplement: Supplementary file 1 — Supplementary Material 1. Table S1. Measurement constructs, indicator codes, and response scale. [file 12889_2026_26667_MOESM1_ESM.docx]

**Table S1. Measurement constructs, indicator codes, and response scale**

| **Construct** | **Indicator codes (as in Table 2)** | **Number of items** | **Response scale** |
| --- | --- | --- | --- |
| Peer influence | PI1-PI4 | 4 | 5-point Likert (1 = Strongly disagree to 5 = Strongly agree) |
| Family support | FS1–FS3 | 3 | 5-point Likert (1 = Strongly disagree to 5 = Strongly agree) |
| Parental monitoring | PM1-PM3 | 3 | 5-point Likert (1 = Strongly disagree to 5 = Strongly agree) |
| Community engagement | CE1-CE3 | 3 | 5-point Likert (1 = Strongly disagree to 5 = Strongly agree) |
| Risk perception | RP1-RP3 | 3 | 5-point Likert (1 = Strongly disagree to 5 = Strongly agree) |
| Self-regulation | SR1-SR4 | 4 | 5-point Likert (1 = Strongly disagree to 5 = Strongly agree) |
| Behavioural intention | BI1-BI3 | 3 | 5-point Likert (1 = Very unlikely to 5 = Very likely) |
| Adolescent drug use | DU1-DU3 | 3 | Frequency scale (0 = Never, 1 = Once, 2 = Occasionally, 3 = Often, 4 = Very often) |

*^*^Indicator codes correspond to those used in the measurement model*
